# Prospective Analysis of Vegetable Amount and Variety on the Risk of All-Cause and Cause-Specific Mortality among US Adults, 1999–2011

**DOI:** 10.3390/nu10101377

**Published:** 2018-09-27

**Authors:** Zach Conrad, Jessica Thomson, Lisa Jahns

**Affiliations:** 1 Grand Forks Human Nutrition Research Center, US Department of Agriculture, Agricultural Research Service, 2420 2nd Ave. N, Grand Forks, ND 58203, USA; lisa.jahns@ars.usda.gov; 2 Delta Human Nutrition Research Program, US Department of Agriculture, Agricultural Research Service, 141 Experiment Station Road, Stoneville, MS 38776, USA; jessica.thomson@ars.usda.gov

**Keywords:** vegetable, variety, index, cardiometabolic, cardiovascular, heart disease, mortality, survival

## Abstract

The Dietary Guidelines for Americans 2015–2020 (DGA) provides recommendations for consuming a specific amount and variety of vegetables, but no studies have assessed the relationship between DGA-recommended vegetable variety and risk of mortality. We prospectively assessed the relationship between vegetable amount and variety and the risk of mortality using nationally-representative survey data (*n* = 29,133). Hazard ratios were estimated using survey-weighted, multivariate, Cox-proportional hazards models. Mean follow-up time was 6.5 years (12.8 years maximum). Total deaths from all causes were 2861, which included 829 deaths from cardiometabolic disease (556 coronary heart disease, 170 stroke, and 103 diabetes). Compared to individuals who reported consuming the greatest amount of vegetables daily, those with the least intake had a 78% greater risk of mortality from all causes (HR: 1.78, 95% CI: 1.29–2.47), a 68% greater risk of death from cardiovascular disease (1.68, 1.08–2.62), and an 80% greater risk of death from coronary heart disease (1.80, 1.09–2.08). No relationships were observed between vegetable variety and risk of all-cause or cause-specific mortality. Greater vegetable amount, but not variety, was associated with a reduced risk of mortality from all causes, cardiovascular disease, and coronary heart disease. Additional large-scale longitudinal studies with repeated measures of dietary exposure are needed.

## 1. Introduction

Greater vegetable intake is associated with a reduced risk of cardiometabolic disease (CMD) [1,2,3,4]. This etiology is largely driven by the bioactive compounds found in vegetables [5], such as carotenoids, polyphenols, folate, and potassium, which are associated with reduced blood pressure, inhibited platelet aggregation, improvement of lipoprotein profiles, increased insulin sensitivity, and reduced oxidant stress and inflammation [6,7,8,9]. Increasing vegetable intake may also displace foods with pro-inflammatory characteristics, such as those high in saturated fat [10].

Yet the health-promoting bioactive compounds found in vegetables are not distributed uniformly across vegetable subgroups (dark green vegetables, red and orange vegetables, legumes, starchy vegetables, and other vegetables) [6]. Therefore, the Dietary Guidelines for Americans 2015–2020 (DGA) provides specific recommendations for consuming a variety of vegetables [11], in order to increase the likelihood that individuals consume adequate amounts of a wide array of health-promoting bioactive compounds.

Bhupathiraju, et al. reported that the number of distinct fruits and vegetables consumed is not associated with a reduced risk of incident coronary heart disease (CHD) [12], yet recently, we demonstrated that greater DGA-recommended vegetable variety is positively associated with prevalent CHD [13]. However, to date, no longitudinal studies have evaluated the relationship between adherence to the DGA vegetable variety recommendations and risk of mortality, to the best of our knowledge. This represents a critical research gap because clinicians use the DGA to provide evidence-based dietary guidance for their patients, but it is not known whether these recommendations are associated with favorable health outcomes.

To address this research gap, we prospectively investigated the relationship between vegetable amount and DGA-recommended variety and the risk of all-cause and cause-specific cardiometabolic mortality among a sample of nearly 30 thousand US adults using data from the National Health and Nutrition Examination Survey (NHANES; 1999–2010) linked to mortality data from the Public-use Linked Mortality Files (1999–2011).

## 2. Materials and Methods

### 2.1. Dietary Data

Data on individual-level demography and health behaviors were acquired from NHANES, cycles 1999–2000, 2001–2002, 2003–2004, 2005–2006, 2007–2008, and 2009–2010 (*n* = 29,133) [14]. NHANES is a cross-sectional survey based on multi-stage probability sampling that collects data on health behaviors and other characteristics from a sample of ~5,000 individuals per year. What We Eat In America (WWEIA) is the dietary component of NHANES. Trained interviewers administer 24-h dietary recalls to study participants using United States Department of Agriculture’s (USDA) Automated Multiple Pass Method [15]. To standardize reported intake amounts across different vegetable types, the MyPyramid Equivalents Database (MPED) and the Food Patterns Equivalents Database (FPED) provide dietary data from WWEIA converted to cup-equivalents. Data on daily vegetable intake (including juice) were acquired from MPED 1.0 (applies to WWEIA 1999–2000 and 2001–2002) and 2.0 (applies to WWEIA 2003–2004); and FPED 2005–2006, 2007–2008, 2009–2010 [16].

### 2.2. Outcome Ascertainment

Dietary data from WWEIA were linked to mortality data from the Public-use Linked Mortality Files (1999–2011) [17], which provide a follow-up for our sample through 31 December 2011, the latest date available. We defined follow-up as the time from WWEIA data collection to death or 31 December 2011 (whichever came first). Deaths were adjudicated by the National Center for Health Statistics (NCHS) using standardized procedures. NCHS staff screen respondents from WWEIA for identifying information such as social security number, name, date of birth, and state of residence, and use probabilistic matching methods to link these respondents to records in the National Death Index (NDI) [18]. The NDI is a database of all US deaths catalogued since 1979, and captures nearly all deaths (~97%) when social security numbers are available, which is the case for all eligible WWEIA respondents linked to the NDI [19,20].

Mortality data were collected for all causes, CHD (International Classification of Disease 10th revision codes I00–I09, I11, I13, I20–I51), stroke (I60–I69), and diabetes (E10–E14). Primary outcomes were deaths from all causes, cardiometabolic disease (CMD; sum of deaths from CHD, stroke, and diabetes), and cardiovascular disease (CVD; sum of deaths from CHD and stroke). Deaths from stroke and diabetes were evaluated independently, but were not included in the final analyses because of small sample sizes and unreliable variance estimates.

### 2.3. Measuring Vegetable Variety

Vegetable variety was measured using an index developed to measure adherence to the DGA-2015 recommendations for daily vegetable variety [13]. The index measures the variety of vegetable intake independent of amount, and penalizes the consumption of vegetable subgroups (dark green vegetables, red and orange vegetables, legumes, starchy vegetables, and other vegetables) that do not align with recommended intake proportions in the DGA 2015–2020 [13]. The index contains two parts. The first part, the Berry Index, assesses the proportionality of vegetable subgroups that individuals report consumed. The consumption targets of DGA 2015–2020 are in weekly units (i.e., cup-equivalents per week), so to be consistent with how WWEIA data are measured, these were converted to daily units. The minimum score (0) represents zero vegetable intake and the maximum score (0.8) represents equal proportions of all vegetable subgroups. The Berry Index can be calculated by

(1) $$\;Berry\;Index=\left(1-\sum o_{i}^{2}\right)\;$$

where *o_i_* is the observed proportion of each vegetable subgroup. The second part of the vegetable variety index, the Health Value [21], gives greater weighting to vegetable subgroups that are recommended in greater proportions. The minimum score (0) represents zero vegetable intake, and the maximum score (1) represents the consumption of only the subgroups with the greatest weights. The Health Value can be calculated by

(2) $$\;Health\;Value=\left(\frac{\sum r_{i}\times o_{i}}{o_{max}}\right)\;$$

where *r_i_* is the recommended proportion of each vegetable subgroup and *o_max_* is the maximum observed proportion out of all vegetable subgroups. Finally, the vegetable variety index is calculated by multiplying the Berry Index by the Health Value, which affirms that higher scores are attained by (1) the consumption of a greater number of vegetable subgroups, and (2) a greater consumption of subgroups that have greater recommended consumption amounts. Index scores range from 0.0 to 0.64.

### 2.4. Statistical Analyses

Vegetable variety score and energy-adjusted total vegetable intake were initially categorized into amount/score quintiles. However, preliminary analyses indicated that different quintiles had similar survival curves, and individuals with zero intake had a unique survival curve. Therefore, for each main exposure variable (vegetable amount and variety), respondents were categorized into three groups: zero intake amount/score, <median intake amount/score, and ≥median intake amount/score (median amount = 1.59 cup-equivalents; median variety score = 0.38). The effects of vegetable amount and variety on the risk of all-cause and cause-specific mortality were estimated using Cox proportional hazards models, which is the most appropriate statistical method to use when assessing time-to-event data, especially when censoring is involved [22]. Time at risk occurred from the date of WWEIA survey administration until death or censoring on December 31st, 2011. The proportional hazards assumption was verified graphically and by an analysis of Schoenfeld residuals. 

Respondents with prevalent cardiometabolic disease during baseline assessments were excluded from cause-specific analyses. All models initially included covariates for age, sex, body mass index (BMI (kg/m^2^)), race/ethnicity (non-Hispanic white, non-Hispanic black, Mexican-American), education (<high school, high school or equivalent, some college, college graduate), income-to-poverty ratio (<0.75, 0.75–1.24, 1.25–1.99, 2.0–3.99, ≥4.00), smoking status (not current, current, unknown), cardiometabolic medication use (yes, no), intake of fatty acids (unsaturated:saturated), and intake of added sugar. Intake of fatty acids and added sugar were energy-adjusted to 2,200 kcal/day using the residual method [23]. To develop a parsimonious final model, non-significant (*p* > 0.05) covariates were iteratively removed. Due to the multiple comparisons in each outcome category, a Bonferroni correction factor was applied, resulting in an *a priori* conservative significance level set at *p* < 0.025.

Stata 15 (StataCorp, College Station, TX, USA) was used for data management, and SAS 9.4 (SAS Institute, Cary, NC, USA) was used for analysis. All analyses accounted for the complex sampling design and sample weights of WWEIA data.

## 3. Results

The mean age of respondents was 46 years, and approximately half (52%) were female. Most respondents were non-Hispanic white (79%) and approximately half (55%) completed at least some college. Approximately two-thirds had an income-to-poverty ratio of at least 2.0 (66%) and a BMI of at least 25 (68%). Just over half (52%) of respondents did not report current smoking status, but of those who did, a similar proportion reported not currently smoking (25%) and currently smoking (24%). Over two-thirds (70%) of respondents reported not being currently prescribed cardiometabolic medication (Table 1).

Over a mean of 6.5 years of follow-up (12.8 years maximum), 2861 deaths were observed from all causes, including 829 deaths from CMD (556 CVD, 170 stroke, and 103 diabetes (Table 1)).

Approximately 5% of individuals (*n* = 1393) reported zero vegetable consumption (least amount group; Table 2). Those in the intermediate intake group (*n* = 13,870) reported a mean intake of 0.89 cup-eq./day (95% CI: 0.88–0.89 cup-eq./day), and those in the greatest intake group reported a mean intake of 2.90 cup-eq./day (2.86–2.93 cup-eq./day).

Approximately 8% of individuals (*n* = 2339) had a daily vegetable variety score of zero (least variety group), meaning they reported consuming up to (and including) one vegetable subgroup (Table 2). Those in the intermediate group (*n* = 13,398) had a daily vegetable variety score of 0.25 (0.25–0.25), and those in the greatest intake group had a daily vegetable variety score of 0.48 (0.48–0.48).

**Figure 1:**
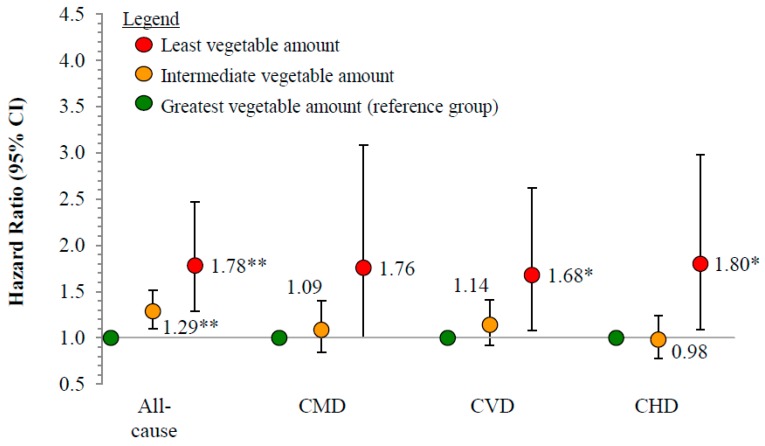
Risk of all-cause and cause-specific cardiometabolic mortality by vegetable intake amount, 1999–2011 (*n* = 29,133). Reference group is individuals consuming the greatest vegetable amount (mean = 2.90 cup-eq./day, 95% CI = 2.86–2.93 cup-eq./day); intermediate: 0.89 cup-eq./day (0.88–0.90 cup-eq./day); least: 0 cup-eq./day. CMD, cardiometabolic disease (CVD + diabetes); CVD, cardiovascular disease (CHD + stroke); CHD, coronary heart disease; ICD-10 codes: Coronary heart disease (I00–I09, I11, I13, I20–I51), stroke (I60–I69), diabetes (E10–E14); Cox proportional hazards model, adjusted for age, sex, race/ethnicity, education, smoking status, cardiometabolic medications, income-to-poverty ratio, body mass index, intake of added sugars, and intake of unsaturated-to-saturated fatty acid ratio. * Significantly different than individuals consuming the greatest vegetable variety for the same event at *p* < 0.05 (Bonferroni adjusted). 95% CIs not adjusted for multiple comparisons. ** Significantly different than individuals consuming the greatest vegetable variety for the same event at *p* < 0.01 (Bonferroni adjusted). 95% CIs not adjusted for multiple comparisons.

Compared to individuals who reported consuming the greatest amount of vegetables daily (Figure 1), those with an intermediate intake had a 29% greater risk of death from all causes (HR: 1.29, 95% CI: 1.10–1.51), and those with the least intake had a 78% greater risk of mortality from all causes (1.78, 1.29–2.47). Individuals with the least intake also had a 68% greater risk of death from CVD (1.68, 1.08–2.62) and an 80% greater risk of death from CHD (1.80, 1.09–2.08), compared to individuals with the greatest intake.

**Figure 2:**
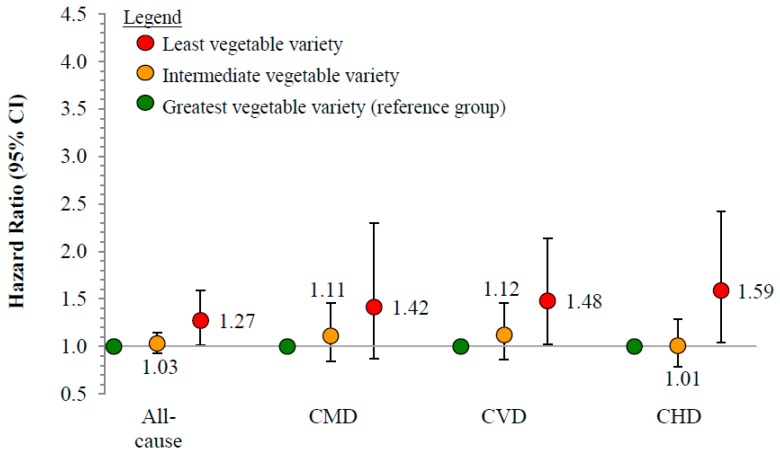
Risk of all-cause and cause-specific cardiometabolic mortality by vegetable intake variety, 1999–2011 (*n* = 29,133). Reference group is individuals consuming the greatest vegetable variety (mean score = 0.48, 95% CI = 0.48–0.48); intermediate: 0.25 (0.25–0.25); least: 0. CMD, cardiometabolic disease (CVD + diabetes); CVD, cardiovascular disease (CHD + stroke); CHD, coronary heart disease; ICD-10 codes: Coronary heart disease (I00–I09, I11, I13, I20–I51), stroke (I60–I69), diabetes (E10–E14); Cox proportional hazards model, adjusted for age, sex, race/ethnicity, education, smoking status, cardiometabolic medications, income-to-poverty ratio, body mass index, intake of added sugars, and intake of unsaturated-to-saturated fatty acid ratio.

No significant relationships were observed between vegetable variety score and the risk of all-cause or cause-specific mortality (Figure 2).

## 4. Discussion

This is the first study to prospectively examine the relationship between daily vegetable variety, as defined by the DGA, and mortality in the US adult general population. To provide important context to vegetable consumption patterns, we also assessed the relationship between vegetable amount and mortality. In this longitudinal analysis of nearly 30 thousand adults followed for up to 12.8 years, we observed that a greater vegetable amount, but not DGA-recommended variety, was associated with a modestly reduced risk of death from all causes, CVD, and CHD.

Recent meta-analyses of prospective cohort studies representing diverse international populations have demonstrated an inverse relationship between vegetable intake amount and risk of mortality from CVD and all causes [2,4]. However, given the limited number of US studies available, these meta-analyses included only one study that used data from the general US population, which relied on data generated from 1971–1992 [24]. Until now, current analyses representing the general US population were lacking. The present study fills this gap by providing the most contemporary prospective analysis of the relationship between vegetable amount and risk of mortality from all causes and cardiometabolic disease subtypes.

Others have reported that greater fruit and vegetable variety was not associated with a reduced risk of incident coronary heart disease [12]; yet variety was measured using a food frequency questionnaire with a restricted number of vegetable choices, which is mentioned by the authors as a limitation. Additionally, vegetable variety was measured as the number of distinct fruits and vegetables consumed rather than adherence to DGA-recommended vegetable variety, and the risk of death was not evaluated [12]. Therefore, the present study fills these important research gaps by prospectively evaluating the relationship between DGA-recommended vegetable variety and the risk of mortality in the US adult general population. 

Although vegetable variety was not independently associated with risk of mortality, it has been demonstrated that increasing vegetable variety is an effective strategy to increase vegetable intake [25]. Previous studies have shown a strong relationship between vegetable variety and amount [13,25,26,27]. A greater variety of vegetables in the diet can increase liking [28], possibly through decreased habituation, which can increase the overall amount consumed [25,26,27]. This suggests that the recommendations to increase vegetable amount and variety remain important clinical and public health messages.

The limitations of this study include the assumption that the exposures (vegetable amount and variety) acted directly on the baseline hazard function and not on the failure time, and that the exposures remained constant over time. Individuals may change their diets over time, and the lack of repeated measures of dietary exposure may have resulted in misclassification. Additional misclassification could have resulted from converting daily intakes to weekly intakes to be consistent with how vegetable variety recommendations are provided in the DGA 2015–2020, and this may also have resulted in an underestimate of vegetable variety scores because individuals are less likely to consume greater variety over the course of a single day than over the course of a week. In addition, we cannot rule out measurement error due to individuals over-reporting the consumption of perceived healthy foods like vegetables, although self-reported data remain useful for comparing dietary patterns between groups [29]. Comparatively wide confidence intervals were observed for the least amount/variety group compared to the intermediate and greatest amount/variety groups, likely due to relatively small sample sizes, so these results should be interpreted cautiously; moreover, the present study, as well as many prospective cohort studies, is limited in its ability to assess causation, thus warranting further caution when interpreting these results. Additional longitudinal studies with repeated measures of dietary exposure are needed to provide support for the observed relationship between vegetable amount and the risk of cardiometabolic mortality.

Vegetables are rich sources of cardioprotective bioactive compounds, including dietary fiber, carotenes, lycopene, nitrate, polyphenols, flavonoids, folate, and potassium [5,6,30], which contribute to weight control, improvement of lipoprotein profiles, reduced blood pressure, inhibited platelet aggregation, increased insulin sensitivity, and reduced oxidant stress and inflammation [7,8,9]. Accordingly, the DGA recommends specific amounts for several subgroups, as well as total vegetables, and a strength of this study includes its novel measurement of vegetable variety, which was designed to assess adherence to specific DGA recommendations. This is the first research, to the best of our knowledge, to examine the longitudinal relationship between DGA-recommended variety and mortality. Additionally, the large sample size and national representativeness of this study make our findings generalizable to the US adult population.

## 5. Conclusions

In this prospective analysis of nearly 30 thousand US adults followed for up to 12.8 years, we observed that a lower vegetable amount, but not DGA-recommended vegetable variety, is associated with a greater risk of all-cause and cause-specific mortality. This is the first prospective study, to the best of our knowledge, to link the concept of vegetable variety to the DGA 2015–2020 vegetable subgroups, which may have stronger implications for clinical practice guidelines than other variety measures. Limitations of this study include the use of baseline dietary intake only, so appropriate caution should be used when interpreting these results. We emphasize that additional longitudinal studies with repeated measures of dietary exposure are needed to more rigorously test the relationship between vegetable variety and risk of CMD mortality. 

## Figures and Tables

**Table 1:** Characteristics of study population.

Characteristic	*n* ^1^	Percent (95% CI) ^2^
No. of deaths		
All-cause	2861	100.0
Coronary heart	556	19.4
Stroke	170	5.9
Diabetes	103	3.6
Cardiovascular	726	25.4
Cardiometabolic	829	29.0
Age (year)	29,133	46.3 (45.8–46.7)
Sex	29,133	
Women		52.2 (51.6–52.8)
Men		47.8 (47.2–48.4)
Race/ethnicity	26,034	
Non-Hispanic white		79.1 (76.9–81.2)
Non-Hispanic black		12.3 (10.9–14.0)
Mexican-American		8.5 (7.2–10.1)
Education	29,088	
Less than high school		19.4 (18.4–20.5)
High school or equivalent		25.3 (24.2–26.3)
Some college		30.2 (29.3–31.0)
College graduate		25.2 (23.6-26.8)
Income-to-poverty ratio	26,770	
<0.75		8.5 (7.9–9.2)
0.75–1.24		11.3 (10.4–12.2)
1.25–1.99		14.5 (13.7–15.3)
2.00–3.99		29.5 (28.4–30.6)
4.00+		36.2 (34.4–38.0)
BMI (kg/m^2^)	28,864	
>18.5		1.6 (1.4–1.9)
18.5–24.9		30.5 (29.5–31.5)
25 to <30		52.6 (51.7–53.5)
≥30		15.2 (14.5–16.0)
Current smoker	29,133	
No		24.7 (23.8–25.6)
Yes		23.8 (22.8–24.7)
Missing		51.6 (50.3–52.9)
Currently prescribed cardiometabolic medication	29,133	
No		70.1 (69.0–71.3)
Yes		29.9 (28.7–31.0)

BMI, body mass index. ^1^ Sample sizes are unweighted. ^2^ Percentages within each column adjusted for survey weight.

**Table 2:** Daily vegetable amount and variety among US adults, 1999–2010.

Vegetable Amount/Variety	Mean (95% CI)		
	Least Intake	Intermediate Intake	Greatest Intake
Amount (cup-eq./day) ^1^	0.00	0.89 (0.88–0.90)	2.90 (2.86–2.93)
Variety (score) ^2,3^	0.00	0.25 (0.25–0.25)	0.48 (0.48–0.48)

^1^ Sample size for low; medium; and high vegetable amount groups = 1393; 13,870; and 13,870; respectively. ^2^ Sample size for low; medium; and high vegetable variety groups = 2339; 13,398; and 13,398; respectively. ^3^ Maximum possible variety score is 0.64.
